# Precocious cell differentiation occurs in proliferating cells in leaf primordia in Arabidopsis *angustifolia3* mutant

**DOI:** 10.3389/fpls.2024.1322223

**Published:** 2024-04-16

**Authors:** Kazune Ezaki, Hiroyuki Koga, Noriko Takeda-Kamiya, Kiminori Toyooka, Takumi Higaki, Shingo Sakamoto, Hirokazu Tsukaya

**Affiliations:** ^1^ Department of Biological Sciences, Graduate School of Science, The University of Tokyo, Bunkyo-ku, Tokyo, Japan; ^2^ Technology Platform Division, Mass Spectrometry and Microscopy Unit, RIKEN Center for Sustainable Resource Science, Yokohama, Kanagawa, Japan; ^3^ Faculty of Advanced Science and Technology, Kumamoto University, Chuo-ku, Kumamoto, Japan; ^4^ International Research Organization for Advanced Science and Technology, Kumamoto University, Chuo-ku, Kumamoto, Japan; ^5^ Bioproduction Research Institute, National Institute of Advanced Industrial Science and Technology, Tsukuba, Ibaraki, Japan

**Keywords:** Arabidopsis, leaf development, cell size, cell proliferation, cell differentiation

## Abstract

During leaf development, the timing of transition from cell proliferation to expansion is an important factor in determining the final organ size. However, the regulatory system involved in this transition remains less understood. To get an insight into this system, we investigated the compensation phenomenon, in which the cell number decreases while the cell size increases in organs with determinate growth. Compensation is observed in several plant species suggesting coordination between cell proliferation and expansion. In this study, we examined an Arabidopsis mutant of *ANGUSTIFOLIA 3* (*AN3*)/*GRF-INTERACTING FACTOR 1*, a positive regulator of cell proliferation, which exhibits the compensation. Though the AN3 role has been extensively investigated, the mechanism underlying excess cell expansion in the *an3* mutant remains unknown. Focusing on the early stage of leaf development, we performed kinematic, cytological, biochemical, and transcriptome analyses, and found that the cell size had already increased during the proliferation phase, with active cell proliferation in the *an3* mutant. Moreover, at this stage, chloroplasts, vacuoles, and xylem cells developed earlier than in the wild-type cells. Transcriptome data showed that photosynthetic activity and secondary cell wall biosynthesis were activated in *an3* proliferating cells. These results indicated that precocious cell differentiation occurs in *an3* cells. Therefore, we suggest a novel AN3 role in the suppression of cell expansion/differentiation during the cell proliferation phase.

## 1 Introduction

Cell proliferation occurs first during organ development in plants, followed by cell expansion and differentiation. The transition timing from cell proliferation to expansion significantly influences the final size of organs that exhibit determinate growth, such as leaves. In *Arabidopsis thaliana* (hereafter, Arabidopsis), all cells first proliferate throughout the leaf primordium, and then the cells cease to proliferate and start cell expansion and differentiation. This transition occurs in a basipetal (from the leaf tip to the base) manner so that distal cells start cell expansion earlier while proximal cells continue proliferating. Finally, cell proliferation activity in the proximal region stops abruptly, and cells rapidly increase in size, leading to rapid expansion of the leaf (Donnelly et al., 1999; White, 2006; Kazama et al., 2010; Andriankaja et al., 2012). Many factors, including transcription factors, kinases, and phytohormones, regulate cell proliferation, expansion, and differentiation. Although several factors have been revealed to be involved in regulating the phase transition, the mechanisms connecting each developmental phase are much less understood (reviewed by Kalve et al., 2014; Vercruysse et al., 2019). The compensation phenomenon, in which the cell number is severely decreased and the cell size is increased in an organ with determinate growth, suggests a coordinated system between cell proliferation and expansion. It has been observed in various species and different types of systems enhance cell expansion, suggesting that several pathways induce compensation (Tsukaya, 2002, 2003, Beemster et al., 2003; Ferjani et al., 2007, reviewed by Horiguchi and Tsukaya, 2011; Hisanaga et al., 2015; Tabeta et al., 2023). One example is the Arabidopsis overexpression line *KIP-RELATED PROTEIN 2* (*KRP2*ox). Excess cell expansion, called compensated cell enlargement (CCE), in *KRP2*ox is caused by increased activity of vacuolar-type H^+^-ATPase (V-ATPase), and cell size increases during the cell proliferation phase (Ferjani et al., 2013). A suppressor mutant of *KRP2*ox compensation is *de-etiolated 3* (*det3*), which has a molecular lesion in the large subunit of V-ATPase (Schumacher et al., 1999; Fukao and Ferjani, 2011; Fukao et al., 2011), but *det3* mutation cannot repress CCE in other compensation-exhibiting mutants (Ferjani et al., 2013). Another example is the *fugu5* mutant, which has a mutation in vacuolar H^+^-pyrophosphatase that leads to deficient gluconeogenesis and fewer cells. The CCE in *fugu5* is induced through auxin biosynthesis, which extends the cell expansion phase (Ferjani et al., 2011; Katano et al., 2016; Takahashi et al., 2017; Tabeta et al., 2021), but this system is not functional in *KRP2*ox compensation. Although several examples of compensation have been identified, only a few mechanisms have been described in detail.

*ANGUSTIFOLIA 3* (*AN3*), also known as *Arabidopsis thaliana GRF-INTERACTING FACTOR 1* (*GIF1*), is a positive regulator of cell proliferation that works together with the SWITCH/SUCROSE NONFERMENTING chromatin-remodeling complex to regulate the expression of target genes. GROWTH REGULATING FACTORs (GRFs) are AN3 interactors, many of which positively regulate cell proliferation. AN3 activates cell proliferation with GRFs and other factors (Kim and Kende, 2004; Horiguchi et al., 2005; Vercruyssen et al., 2014, reviewed by Kim and Tsukaya, 2015; Kim, 2019). In the leaf primordia, AN3 is expressed in the proximal region and diffuses to the distal region, resulting in a graded distribution of active cell proliferation (Kawade et al., 2017). AN3 and GRFs are negatively regulated by TEOSINTE BRANCHED 1/CYCLOIDEA/PROLIFERATING CELL NUCLEAR ANTIGEN FACTOR 4 (TCP4). TCP4 also produces microRNA miR396, which targets seven of nine *GRF*s and decrease their expression levels (Liu et al., 2009; Rodriguez et al., 2010; Wang et al., 2011). In a developing leaf primordium, the level of miR396 increases along the proximal-distal axis and accumulates more in the distal part. It makes *GRF*s expression exhibit the opposite pattern (*i.e.*, higher in the proximal and lower in the distal) and leads to lower cell proliferation activity in the distal part (Rodriguez et al., 2010). By introducing miRNA-resistant version of *GRF*s, the graded pattern of GRF distribution disappear and cell proliferation activity is maintained for longer time (Rodriguez et al., 2010; Debernardi et al., 2014). Thus, AN3-GRF module is an important factor to regulate cell proliferation activity.

The *an3* mutants in several plant species, including Arabidopsis, *Oryza sativa* (rice), and *Zea mays* (maize), exhibit compensation (hereafter, *an3*-mediated compensation; Horiguchi et al., 2005; Shimano et al., 2018; Zhang et al., 2018) with severe decrease in leaf cell proliferation. For example, in Arabidopsis *an3* mutants, leaf cell number decreases to approximately 30% and cell size increases to approximately 150% compared to that of the wild type (Horiguchi et al., 2005). Previous studies have shown several features of *an3*-mediated compensation: the cell expansion rate in *an3* is distinctively increased (Ferjani et al., 2007; Lee et al., 2009); ribosome biogenesis- or activity-related mutations synergistically enhance *an3*-mediated compensation (Fujikura et al., 2009); *an3* CCE is ploidy-independent process and is suppressed by e*xtra-small sister 2* mutant, in which salicylic acid signaling is highly activated (Fujikura et al., 2007, 2020); *an3* CCE occurs non-cell-autonomously in palisade tissue cells and cell-autonomously in epidermal cells (Kawade et al., 2010; Nozaki et al., 2020). However, the detailed mechanism of *an3*-mediated compensation, particularly the cause of *an3*-mediated CCE has not yet been elucidated.

During leaf development, the cell size is regulated differently during the proliferation and expansion phases. In the cell proliferation phase, cells repeatedly undergo cell division to maintain a constant cell size. A constant cell size in proliferating cells is a universal phenomenon called cell-size homeostasis, which is observed in bacteria, animals, and plants. Several mechanisms have been proposed to explain how cells maintain constant size (Schaechter et al., 1962; Fantes, 1977; Johnston et al., 1977; Campos et al., 2014; Ginzberg et al., 2015). In plants, cell-size homeostasis is often investigated in the shoot apical meristem cells. A specific factor other than KRP4, which is inherited on the chromatin during mitosis, has not yet been identified. KRP4 is an indicator of DNA content and inhibits cell division until it is sufficiently diluted during cell growth (D’Ario et al., 2021). As seen in shoot apical meristem cells, proliferating cells in developing leaf primordia maintain a constant size (Donnelly et al., 1999; De Veylder et al., 2001; Andriankaja et al., 2012). Long-term live cell imaging showed that these cells slightly increase in size just before the onset of the cell expansion phase, which occurred concurrently with an extended cell cycle length (Fox et al., 2018).

After the onset of the cell expansion phase, cells rapidly increase in size through several processes, such as vacuole development and changes in the mechanical properties of the cell wall (reviewed by D’Ario and Sablowski, 2019). The process of vacuole development has been well-documented in root cells (Viotti et al., 2013; Cui et al., 2019). In meristematic cells, several small vacuoles are distributed in a cell and gradually fuse to increase the total volume and occupancy of the cell. Fused vacuoles become a large central vacuole which is required for water uptake and increase of the cell size (Cui et al., 2019). Cell wall loosening is another factor that increases cell size in response to increased turgor pressure (Cosgrove, 2005). Several cell wall modification proteins, such as expansins and xyloglucan endotransglucosylases/hydrolases, and the methylesterification of pectins, such as homogalacturonan, change cell wall rigidity in both directions to increase or reduce stiffness. Pectin demethylesterification triggers cell-wall loosening during organ development in the shoot apical meristem and hypocotyl, as well as cell shape changes in leaf pavement cells (Cosgrove, 2005; Peaucelle et al., 2008, 2011, 2015, Haas et al., 2020; Wang et al., 2020, reviewed by Wolf and Greiner, 2012). In addition, chloroplast development has been suggested to be associated with the onset of cell expansion. Andriankaja et al. (2012) showed that chloroplast development and upregulation of photosynthesis-related genes occur just before the onset of cell expansion in developing Arabidopsis leaf primordia. They treated developing leaf primordia with norflurazon, a chemical inhibitor of chloroplast differentiation, resulted in delayed cell expansion onset. This suggests that chloroplast development and retrograde signaling are important triggers for the onset of cell expansion.

In this study, we examined the *an3*-mediated compensation mechanism, focusing on early-stage leaf primordia, the timing *AN3* is expressed in wild type. We conducted kinematic, cytological, biochemical, and transcriptome analyses, and the results suggested that *an3* cells exhibit precocious cell expansion and differentiation during the cell proliferation phase.

## 2 Materials and methods

### 2.1 Plant materials and growth conditions

The wild-type accession was Columbia-0 (Col-0). The seeds of Col-0 and *an3-4* were provided by Dr. Kensuke Kawade (Saitama University), and *an3-2* from Dr. Ushio Fujikura (Japan Agency for Medical Research and Development). The original description of *an3-2* and *an3-4* was provided by Horiguchi et al. (2005) *an3-2* has a 6-base deletion in the SNH domain and *an3-4* is a null allele having a large deletion. Seeds were sown on rockwool (Nittobo, Tokyo, Japan or Grodan, Roermond, Netherlands), watered daily with 0.5 g L^-1^ Hyponex (Hyponex, Osaka, Japan), and grown under white fluorescent lamps (50–90 μmol m^-2^ sec^-1^) at 22°C, in a long-day photoperiod of 16-hour light/8-hour dark.

### 2.2 Histological observation

Seedlings were fixed in 4% (w/v) paraformaldehyde solution containing 0.05% (v/v) Triton-X100 using vacuum desiccator or centrifugation to measure the leaf size and adaxial subepidermal cell size in the first foliage leaf primordia. After fixation, the samples were washed with phosphate-buffered saline (PBS) and cleared using ClearSee (Kurihara et al., 2015) containing 1% (v/v) Calcofluor White Stain (Sigma-Aldrich, Saint Louis, MO, USA) to stain the cell wall (the final calcofluor white concentration was 10 μM). For observation of nuclei, SYBR Green I Nucleic Acid Gel Stain (Lonza, Basel, Switzerland) was added to ClearSee at a 2000-fold dilution. Leaf primordia were cut under a stereomicroscope and observed under a confocal microscope (FV3000, Olympus, Tokyo, Japan or FV10i, Olympus). Calcofluor was excited with a laser at 405 nm, emission was detected between 430 and 470 nm, and SYBR Green was excited at 488 nm and emission was detected between 490 and 540 nm. The measurements were performed using ImageJ (Schneider et al., 2012; RRID: SCR_003070) or Fiji (Schindelin et al., 2012; RRID: SCR_002285). Leaf and cell shape were traced manually or by using ‘Morphological Segmentation’ plugin (Legland et al., 2016), and the area was measured using the “Analyze particles” function.

### 2.3 Kinematic analyses

Adaxial subepidermal cells were measured in the kinematic analyses. For early small leaf primordia (from 3 to 5 DAS), the cell area was measured using the Volume Viewer plugin in Fiji to make optical sections parallel to the adaxial surface. Proximal and distal cells were measured at approximately 25% and 75% of the leaf blade length, respectively, as described by De Veylder et al. (2001). For 3 and 4 DAS leaf primordia, cell area was measured at around 50% of the leaf blade length because the leaf was too small to measure proximal/distal cell area separately. For leaves on 5 DAS afterwards, average cell area was calculated by taking the average of proximal and distal cell area. The number of cells per leaf was estimated by dividing the leaf size by the average cell area. Statistical analyses were performed using R software.

### 2.4 Cytological observation by FE-SEM

The 4 DAS seedlings were fixed with 0.05 M cacodylic acid buffer containing 4% paraformaldehyde and 2% glutaraldehyde in a vacuum for 40 min and kept at 4°C overnight. After post-fixation with 1% (w/v) osmium tetroxide, samples were dehydrated with a graded methanol series, embedded in TAAB Epon812 resin (Nisshin EM Co., Ltd., Tokyo, Japan), and sectioned at 1 μm thickness. Sections on a glass slide were stained with 0.4% uranyl acetate and lead citrate solution and coated with osmium using an osmium coater (HPC-1SW, Vacuum Device, Ibaraki, Japan). Cross-sections around the 50% of the leaf blade length were observed using a field emission scanning electron microscope (FE-SEM) (SU8220, Hitachi High-Tech, Tokyo, Japan) with an yttrium aluminum garnet backscattered electron detector at an accelerating voltage of 5 kV. Cell wall thickness was measured in adaxial subepidermal cells where a pair of facing plasma membranes was clear. Three to five locations were measured for each individual, and a minimum value was obtained from 20 measurements for each part (n = 3 individuals for the wild type and *an3*).

### 2.5 Vacuole observation and measurement

Vacuoles were observed in the first foliage leaf primordia at 5 DAS. The vacuoles and plasma membranes were stained with BCECF-AM (Thermo Fisher Scientific, Waltham, MA, USA) and FM4-64 (Thermo Fisher Scientific), respectively. A basal buffer [50 mM KCl, 5 mM 2-(N-morpholino)-ethanesulfonic acid (MES)-Tris (pH 6.5), and 10 mM CaCl_2_] (Higaki et al., 2012) was used as a solvent to minimize the osmotic change. After one of the cotyledons was cut, the seedlings were placed in a staining solution and kept in the dark for 2 h at approximately 22°C. After staining, foliage leaf primordia were dissected and mounted with basal buffer for observation under a confocal microscope (FV3000, Olympus). BCECF was excited with a laser at 488 nm, and the emission was detected between 500 and 570 nm. For FM4-64, the excitation wavelength was 514 nm, and emission was detected between 600 and 640 nm.

Image analysis was conducted using Fiji software. For cell segmentation, we used 1.5-pixel Gaussian-filtered images of plasma membranes labeled using FM4-64 and the ‘Morphological Segmentation’ plugin (Legland et al., 2016). Segmentation was performed with a tuned tolerance value that was adjusted depending on the image. For vacuole segmentation, we used images of vacuoles labeled with BCECF. The BCECF images were subjected to a bandpass filter (3–18 pixels), and then they were manually binarized. Finally, we measured the cell and vacuole areas using the ‘Analyze particles’ function. The volumes were calculated based on the areas and z-stack thickness (0.42 μm).

### 2.6 Cell wall monosaccharide analysis

The 4 DAS first-foliage leaf primordia (from Col and *an3-4*, n = 60 individuals, N = 4 replicates) were fixed in 80% (v/v) ethanol. After fixation, samples were treated with methanol three times for 30 min, three times with acetone for 30 min, and three times with methanol/chloroform for 30 min, rinsed twice with ethanol, and dried at 65°C overnight. The dried tissues were hydrolyzed with sulfuric acid and neutralized with calcium carbonate. The neutralized solution was labeled with p-aminobenzoic ethyl ester (ABEE) and analyzed using UPLC (ultra-performance liquid chromatography).

### 2.7 mRNA sequencing analysis in 5 DAS leaf primordia

For RNA isolation, the first foliage leaf primordia at 5 DAS were collected under a stereomicroscope (30–50 individuals per a replicate, three replicates each for wild type and *an3*). The collected samples were immediately frozen in liquid nitrogen and crushed using zirconia beads (TOMY, Tokyo, Japan) and a bead beater (TissueLyser II, Qiagen, Hilden, Germany). RNA was extracted using the FastGene RNA Premium Kit (NIPPON Genetics Co., Ltd., Tokyo, Japan) and modified to use a QIA shredder column (Qiagen) in the first step. Sequencing libraries were prepared using the NEBNext Ultra II Directional RNA Library Prep Kit for Illumina (New England Biolabs, Tokyo, Japan) and the NEBNext Poly(A) mRNA Magnetic Isolation Module (New England Biolabs). Libraries were sequenced on the HiSeqX platform (Illumina, Inc., California, USA) by Macrogen Japan (Kyoto, Japan) to obtain 150 bp paired-end reads. The reads were trimmed and filtered based on quality using fastp (version 0.20.1) (Chen et al., 2018) and then mapped to the public *Arabidopsis thaliana* reference genome (TAIR10) using STAR (version 2.7.7) (Dobin et al., 2013). Read counts were calculated using RSEM (version 1.3.3). Count normalization and DEG detection were performed using the TCC R package (Sun et al., 2013) with EdgeR (Robinson and Oshlack, 2010). The data were visualized using the following packages of the R software: tidyverse (Wickham et al., 2019) and Heatmaply (Galili et al., 2018). The reads were deposited in the DDBJ Sequence Read Archive under the run accession number DRR506123–DRR506128.

## 3 Results

### 3.1 The *an3* mutant exhibited precocious cell enlargement in proliferating cells

**Figure 1:**
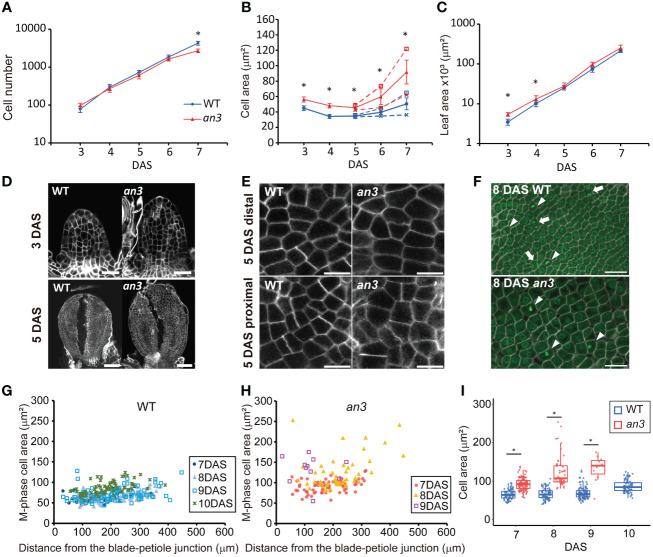
Kinematic analyses of leaf primordia of wild type and the *an3* mutant. **(A–C)** Cell number **(A)**, cell area **(B)**, and leaf area **(C)** from first foliage leaf primordia of WT (circle, blue) and *an3* (triangle, red), 3 to 7 day-after-stratification (DAS). Cell area was measured in the second layer of the adaxial side. In B, the proximal (cross mark) and distal (open square) cell areas are shown separately from 5 DAS onward using dotted lines, and the average cell area in a leaf is shown using solid lines. Between the distal and proximal cell area, a significant difference was seen from 6 DAS but not at 5 DAS in both WT and *an3* (p < 0.01, Wilcoxson rank sum test). Asterisks in B indicate significant differences in average cell area between WT and *an3.* Mean ± SD. n = 180–270 cells from 6–9 individuals were measured (* p < 0.01, Welch’s t-test for **A** and Wilcoxson rank sum test for **B, C**). **(D)** Representative images of 3 DAS and 5 DAS leaf primordia. Note that 5 DAS leaf primordia were cut to make them flat. Bars = 20 μm (3 DAS) and 50 μm (5 DAS). **(E)** Representative images of proximal and distal cells at 5 DAS. Bars = 10 μm. **(F)** M phase cell images in a proximal part of 8 DAS leaves. Cells were stained by calcofluor white (cell wall, white) and SYBR Green (nuclei, green). The M phase cells had one or two condensed nuclei showing strong fluorescent signal with a not round shape (arrowheads and arrows, respectively). Bars = 10 μm. **(G, H)** Area of M-phase cells from 7 to 10 DAS in WT **(G)** or 7 to 9 DAS in *an3* **(H)**, respectively. The X-axis shows the distance from the leaf blade-petiole junction. In total, 11–114 cells from 3–6 leaves were measured for each DAS. **(I)** Box plot of the M-phase cell area in WT (blue) and *an3* (red). The data shown in **(I)** is same as **(G, H)** represented in a different manner. Boxes represent 25th to 75th percentile with a median line. Asterisks indicate significant differences between WT and *an3* on each DAS (* p < 0.01, Wilcoxson rank sum test).

To investigate the mechanism of *an3*-mediated compensation, we focused on the early-stage leaf primordia, in which all cells proliferate. We performed kinematic analyses in the first foliage leaf primordia of wild type and *an3-4* mutant (Horiguchi et al., 2005), which is a null allele of *an3* mutant, to measure the leaf area, the adaxial subepidermal cell area, and estimated cell number (**Figures 1A–E**). The cell size was already larger in the *an3* mutant at 3 DAS (**Figure 1B**) when the leaf primordia still exhibited a cylinder shape (**Figure 1D**). Previous studies of wild type leaf primordia showed that in the early-stage leaf primordia, actively proliferating cells exhibit a constant size throughout a leaf (Donnelly et al., 1999; Andriankaja et al., 2012; Fox et al., 2018). In our kinematic analysis, cell size was relatively constant throughout the leaf primordium in both the wild type and *an3* mutant at 5 DAS (**Supplementary Figure 1**), indicating that actively proliferating cells were enlarged in the *an3* mutant. After 6 DAS, the distal cells started to expand in both wild-type and *an3* plants, with a higher expansion rate in the *an3* mutant (**Figure 1B**). This was consistent with the results of previous studies (Ferjani et al., 2007; Lee et al., 2009). The same tendency of cell size increase in the early stage was confirmed in another *an3* mutant allele, *an3-2* (Horiguchi et al., 2005), which has a 6-base deletion in the SNH domain (**Supplementary Figure 2**). These results indicated that the *an3* mutant had larger cells during the proliferation phase. In addition, we measured cell size in the shoot apical meristem (SAM) and did not detect significant difference between wild type and *an3* (**Supplementary Figure 3**), suggesting that at least after the leaf primordia emergence, the cell size difference start to be appeared.

In the proximal region, the *an3* cells started to further increase in size at 7 DAS, which was earlier than the wild type (**Figure 1B**). This was when the decrease in cell proliferation became apparent (**Figure 1A**), therefore, we checked if those *an3* cells had already lost their cell proliferation ability. To this end, we stained the nuclei and cell walls using SYBR Green and calcofluor white, respectively, to identify cells undergoing mitosis (M phase), which had one or two condensed nuclei with high fluorescent intensity (**Figure 1F**). M phase cells in the *an3* mutant were observed until 9 DAS but not at 10 DAS, whereas dividing cells were still observed at 10 DAS in the wild type (**Figures 1G–I**). In the wild type, the M phase cell size was relatively constant from 7 to 9 DAS and was slightly enlarged at 10 DAS (**Figures 1G, I**), showing an increase in cell size only at the end of the proliferation phase, which is consistent with a previous study (Fox et al., 2018). In contrast, the M phase cells in the *an3* mutant already exhibited much larger and more varied sizes at 7 DAS (**Figures 1H, I**). In addition, cell size increased until 9 DAS, which is the end of the proliferation phase, indicating that cell size increased regardless of proliferation activity. Taken together, the *an3* cells exhibited larger proliferating cells which did not maintain a certain size, and those precociously enlarged *an3* cells exited proliferation earlier than the wild type.

### 3.2 Cytological and biochemical phenotypes indicated precocious cell differentiation in the *an3* mutant

**Figure 2:**
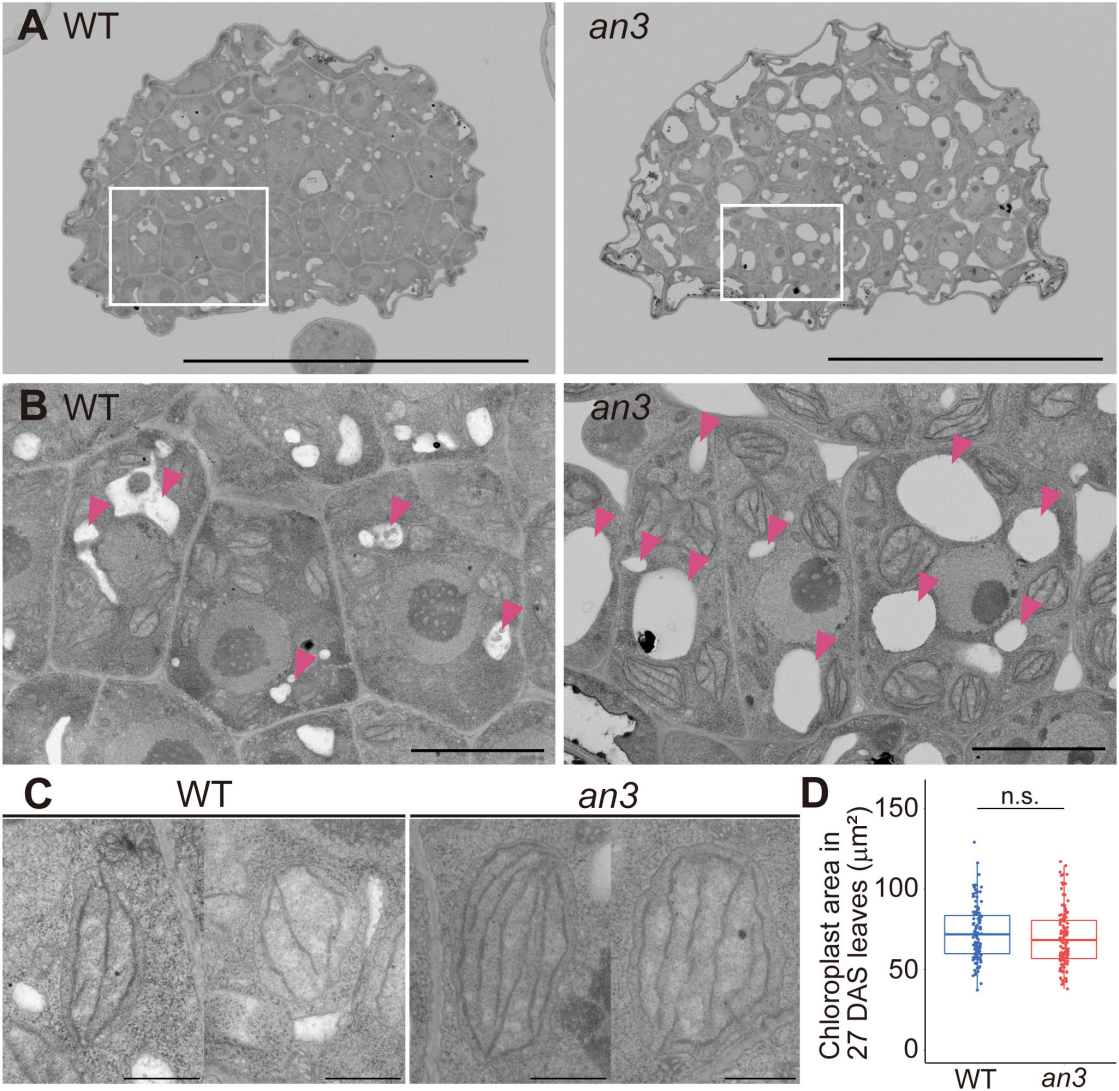
FE-SEM observation of 4 DAS leaf primordia and chloroplast size in mature leaves. **(A)** Cross-section images of central part of 4 DAS leaf primordia taken using FE-SEM. The upper side of an image is the abaxial side and the lower is the adaxial side. **(B)** Magnified view of adaxial subepidermal cells highlighted by rectangles in **(A)**. Arrowheads indicate vacuoles. **(C)** Representative chloroplast images of WT and the *an3* mutant. **(D)** Chloroplast area in mature first leaves (27 DAS) (n = 116 (WT) and 133 (*an3*) chloroplasts from 3 individuals. n.s, not significant; Student’s t-test). Bars = 50 μm **(A)**, 5 μm **(B)**, and 1 μm **(C)**.

Since the *an3* mutant exhibited larger cells regardless of cell proliferation ability, we examined the factors that expand *an3* cells. We performed cytological observations of cross-sections of 4 DAS leaf primordia using a field emission-scanning electron microscope (FE-SEM). Even though cells were actively dividing at this stage, *an3* cells had larger vacuoles and extended intercellular spaces, whereas wild-type cells exhibited only small vacuoles and much less intercellular space (**Figures 2A, B**). In addition, chloroplasts in *an3* cells were more developed with more thylakoid membranes than those in the wild type (**Figure 2C**). It implied that chloroplast development was accelerated precociously in *an3* proliferating cells. In mature leaves, it has been reported that the chloroplast number per cell was increased in *an3* leaves (Kawade et al., 2013). To examine whether the mature chloroplast size was changed in *an3* leaves, we measured the chloroplast size in mature leaves (27 DAS). However, it was not significantly different between wild-type and *an3* cells (**Figure 2D**).

**Figure 3:**
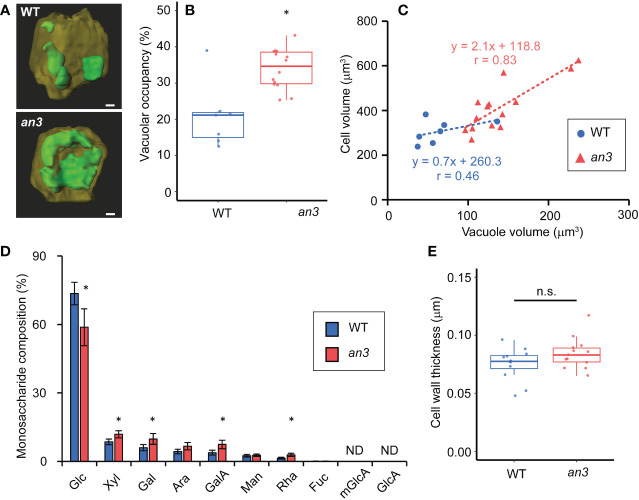
Vacuole volume measurement and cell wall analyses in 4 or 5 DAS leaf primordia. **(A)** Reconstructed 3D images of vacuoles in WT and *an3* cells. Representative images are shown. The vacuole is shown in green, and the other parts of the cell are dark yellow. For the observation, 5 DAS first leaf primordia were stained by BCECF-AM (vacuole) and FM4-64 (plasma membrane). Bars = 10 μm. **(B)** Vacuolar occupancy (the percentage of vacuole volume in cell volume) in WT and *an3* cells (n = 7 (WT) and 14 (*an3*) cells, *p < 0.01, Welch’s t-test). **(C)** Relationship between vacuole volume and cell volume. Regression lines are shown by dotted lines. Pearson’s product-moment correlation coefficient: r = 0.46 (WT) and r = 0.83 (*an3*) (n = 7 (WT) and 14 (*an3*) cells). **(D)** Monosaccharide composition in 4 DAS leaf primordia cell wall. Relative values to total sugar are shown. Glc: glucose, xyl: xylose, gal: galactose, ara: arabinose, galA: D-galacturonic acid, man: mannose, rha: rhamnose, fuc: fucose, mGlcA: 4-O-methyl glucuronic acid, GlcA: glucuronic acid. mGlcA and GlcA were not detected. (* p < 0.05, Student’s t-test). **(E)** Cell wall thickness of WT and *an3* in subepidermal cells measured with FE-SEM images (n = 12 (WT) and 13 (*an3*) parts from 3 individuals. n.s., not significant; Welch’s t-test).

We then measured vacuole volume to determine its effect on cell size. To this end, vacuoles and plasma membranes in living 5 DAS leaf primordia, in which still all cells were proliferating, were stained using BCECF-AM and FM4-64, respectively, and observed using confocal microscopy. The constructed 3D images showed clear differences in vacuole size and shape, which were small and dispersed in the wild type but larger and more unified in the *an3* cells (**Figure 3A**). The vacuolar occupancy, the percentage of vacuole volume in cell volume, was much higher in *an3* cells than in wild type (**Figure 3B**). The fused vacuole in the *an3* cells was similar to that observed in more differentiated wild-type cells (Cui et al., 2019). Moreover, vacuole volume was correlated with cell volume, especially in *an3* cells (**Figure 3C**), indicating that more-developed vacuoles participated in precocious cell enlargement in proliferating *an3* cells.

We also analyzed the cell wall composition of proliferating cells to gain insights into cell wall properties. The relative amounts of monosaccharides in the total sugar were measured in leaf primordia at 4 DAS, in which all cells proliferated. The *an3* mutant contained lower level of glucose and more xylose, galactose, D-galacturonic acid, and D-rhamnose (**Figure 3D**). In *an3*, the relative levels of galactose, D-galacturonic acid, and D-rhamnose, which are pectin components, were higher compared to those in wild type, suggesting that pectin in the intercellular matrix, such as the middle lamella, was increased in the *an3* mutant. Pectin is often associated with cell wall loosening through demethylesterification during the organ formation or cell differentiation (Peaucelle et al., 2008, 2011, 2015, Haas et al., 2020). The changed cell wall composition in *an3* proliferating cells may lead to altered cell wall extensibility. Glucose is a component of cellulose and hemicellulose; therefore, less glucose and more pectin in *an3* would change cell wall characteristics. However, no significant difference was detected in cell wall thickness between the wild type and the *an3* mutant (**Figure 3E**).

The *an3* cells in the proliferation phase exhibited more-developed vacuoles, chloroplasts, and intercellular spaces as well as different cell wall composition. These phenotypes indicated that the *an3* cells differentiated precociously despite their active proliferative activity. Moreover, the precocious cell expansion in the *an3* mutant was at least partially attributed to more fused and developed vacuoles.

### 3.3 Gene expression during the proliferation phase also supported the precocious cell differentiation in *an3*

To examine the altered gene expression profile in the *an3* mutant associated with the precocious onset of cell expansion, we conducted mRNA sequencing (mRNA-seq) analyses using 5 DAS leaf primordia, in which all cells proliferated. In total, 1683 genes were detected as differentially expressed genes (DEGs; 1154 upregulated and 529 downregulated, including *AN3* itself) in the *an3* mutant compared to the wild type (false discovery rate (FDR) < 0.05; **Table S1**). Although many GRFs are AN3-interactors, only *GRF4* and *GRF9* were involved in DEGs that were down- and upregulated, respectively.

**Figure 4:**
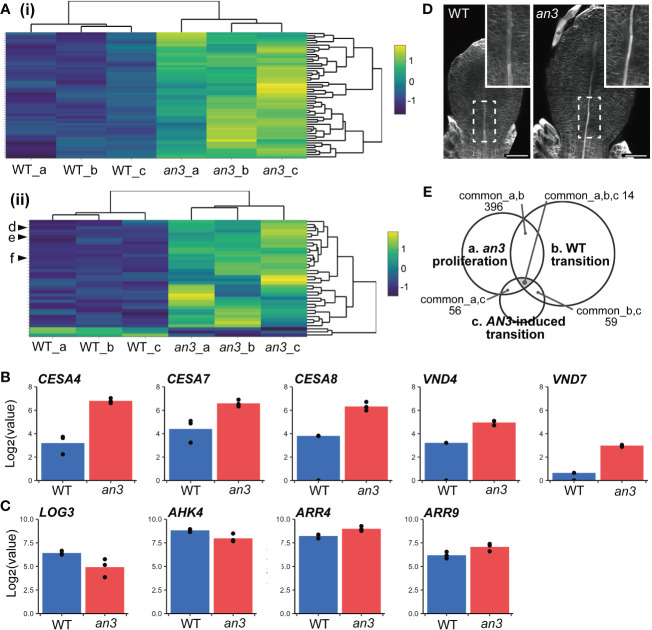
Characterization of DEGs of the *an3* mutant in mRNA-seq analyses. **(A)** Heatmap of *an3* DEGs involved in GO term “photosynthesis” (GO:0015979; 54 of 241 assigned genes; (i) and “plant-type secondary cell wall biogenesis” (GO:0009834; 38 of 158 assigned genes; (ii). Each row represents a gene. Column a, b, and c show replicates. The color code indicates z-score calculated from normalized counts. In (ii), d, e, and f indicate *CESA7*, *CESA8*, and *CESA4*, respectively. **(B, C)** Expression levels of genes involved in secondary cell wall synthesis or vessel formation (*CESA*s and *VND*s; B), and CK biosynthesis or response (*LOG3*, *AHK4*, and *ARR*s; C) in WT (blue) and *an3* (red). Log-transformed normalized counts are shown. Each dot shows replicates. **(D)** 5 DAS leaf primordia of WT and *an3* stained with calcofluor (cell wall). Insets show a magnified view of differentiating xylems indicated by dotted rectangles. It should be noted that cell number was similar between WT and *an3* at this stage, and the leaf size difference was attributed to cell size difference. Bars = 50 μm. **(E)** A Venn diagram of transcriptome data comparison. Common DEG numbers are shown among our data of 5 DAS *an3* leaf primordia containing proliferating cells (a), DEGs in the transition phase in WT leaf primordia (Andriankaja et al., 2012; b), and DEGs in *AN3*-induced leaf primordia containing both proliferating and expanding cells (Vercruyssen et al., 2014; c).

Next, we compared published data on gene expression changes in the wild type during the transition phase from cell proliferation to expansion. We used microarray data from 3rd foliage leaf primordia between days 9 and 10, which corresponds to the timing that distal cells cease proliferation and start expansion (2,208 DEGs; Andriankaja et al., 2012). With *an3* DEGs, 396 genes were found in common. Of these 396 genes, 392 genes showed the same tendency in expression changes (*i.e.*, upregulated or downregulated) in *an3* mutant and in day 10 leaf primordia, while only 4 genes exhibited the opposite pattern (Spearman’s rank correlation: ρ = 0.86; **Supplementary Figure 4A**). It suggested that gene expression changes in *an3* mutant was at least partially similar to those in the more-developed stage leaf primordia. Gene Ontology (GO) term enrichment analysis (Berardini et al., 2004) of the 392 common genes showed that many genes related to photosynthesis were upregulated in *an3* (**Figure 4Ai**). These involved genes encoding chlorophyll-binding proteins and factors in photosystems I and II, suggesting that photosynthetic activity was higher in *an3* cells than that in wild-type cells during the proliferation phase. This was consistent with the FE-SEM observations, which showed more-developed chloroplasts in *an3* cells (**Figure 2C**). In addition, many upregulated genes among the *an3* DEGs were involved in secondary cell wall biosynthesis, such as *CELLULOSE SYNTHASE A4* (*CESA4*), *CESA7*, and *CESA8* (Gardiner et al., 2003; Taylor et al., 2003; **Figure 4Aii**). It would be associated with xylem formation since the master regulators of xylem vessel formation, i.e., *VASCULAR RELATED NAC-DOMAIN 4* (*VND4*) and *VND7* (Kubo et al., 2005; Zhou et al., 2014), were also included in *an3* DEGs and were upregulated in *an3* (**Figure 4B**). This was consistent with the *an3* mutant phenotype showing earlier xylem differentiation than the wild type (**Figure 4D**). These results, together with the cytological observations described above, indicate that *an3* cells exhibited accelerated cell expansion and differentiation processes.

To focus on genes related to precocious cell expansion and differentiation downstream of AN3, we compared microarray data for 164 DEGs from *AN3*-induced leaf primordia that was 8 DAS 1st foliage leaves, 8-hour after the *AN3* induction, which contained both proliferating and expanding cells (Vercruyssen et al., 2014). Among the three transcriptome datasets, 14 genes (6 upregulated and 8 downregulated in *an3*) were common (**Figure 4E**, **Supplementary Figure 5**, **Table S2**). Among these common DEGs, some genes downregulated in *an3* were associated with chromatin or ribosome processing, such as *EBNA1 BINDING PROTEIN 2* and *OLIGOCELLULA 2* (Fujikura et al., 2009; Romanova et al., 2009), both of which are related to the AN3 role in cell proliferation. In contrast, upregulated genes in *an3* did not show any apparent characteristics, except for AT1G68570, named *NRT1/PTR FAMILY 3.1* (*NPF3*) encoding a membrane-localized gibberellic acid (GA) transporter. A previous study has indicated that NPF3 plays a role in GA uptake in elongated endodermal cells in Arabidopsis roots (Tal et al., 2016). Moreover, GA plays a role in regulating the phase transition from cell proliferation to expansion in maize leaf development (Nelissen et al., 2012). By comparing the data sets of *AN3*-induced leaf primordia and wild-type transition-phase leaf primordia, all of 59 common DEGs showed the opposite pattens in gene expression changes (Spearman’s rank correlation: ρ = -0.56; **Supplementary Figure 4B**). It also supported our hypothesis that AN3 has a roll in suppression of cell differentiation. GO term enrichment analysis of these genes showed many terms related to RNA processing and gene expression.

Other studies have shown that cytokinin (CK) deficiency during the cell proliferation phase suppresses cell division and induces precocious cell differentiation, leading to fewer and larger cells (Holst et al., 2011; Skalák et al., 2019). This phenotype was very similar to that in the *an3* mutant. In wild-type leaf primordia, the amount of CKs, especially *trans*-zeatin (*t*Z), dramatically changes, peaking around the transition phase and dropping afterwards. Among CK biosynthesis genes, such as *ISOPENTENYL TRANSFERASE*s (*IPT*s) and *LONELY GUY*s (*LOG*s), only *LOG3* showed decreased expression just before the CK decrease (Skalák et al., 2019). Analogous to this change, no *LOG*s and *IPT*s except for *LOG3* was present in *an3* DEGs, and *LOG3* was downregulated in the *an3* mutant (**Figure 4C**). Among *ARABIDOPSIS HISTIDINE KINASE*s (*AHK*s), the CK receptors, *AHK4* was included in *an3* DEGs and downregulated, and among type-A *ARABIDOPSIS RESPONSE REGULATOR*s (*ARR*s), the CK response genes, *ARR4* and *ARR9* were in *an3* DEGs and upregulated (**Figure 4C**). The expression changes in *AHK4* and *ARR4* were similar to those in wild-type leaf primordia during the transition phase from proliferation to expansion (Skalák et al., 2019).

## 4 Discussion

### 4.1 A novel AN3 role suppressing the onset of cell differentiation during the cell proliferation phase

In this study, we investigated Arabidopsis *an3* mutants to determine the CCE mechanism in *an3*-mediated compensation. We found that the cell size in the *an3* mutant significantly increased while cells were actively dividing. Since *an3* proliferating cells increased in size, the precocious cell expansion and differentiation in *an3* was not a simple shift of the transition timing but an abnormal overlap between the cell proliferation and expansion/differentiation phases. In our cytological observations and RNA-seq analyses, more-developed chloroplasts and vacuoles, earlier xylem development, and accelerated photosynthetic activity were observed in *an3* proliferating cells. These results support the idea that an increase in cell size in the *an3* mutant occurred through precocious cell differentiation, suggesting that AN3 suppresses cell differentiation during the cell proliferation phase.

In the previous studies, AN3 has been identified as a transcriptional coregulator (Vercruyssen et al., 2014; Nelissen et al., 2015; Ercoli et al., 2018). AN3-GRF module has a key role in activating the cell proliferation in the shoot (Horiguchi et al., 2005; Vercruyssen et al., 2014; Nelissen et al., 2015), and another module together with *PLETHORA1* (*PLT1*) and *SCARECROW* regulates the meristem size in the root. In roots, AN3 and other GIFs repression on *PLT1* expression limits the meristem size (Ercoli et al., 2018). Although several different AN3-interactors have been identified, AN3 role is supposed to regulate cell proliferation activity. Here, we propose a novel AN3 role for suppression of cell expansion/differentiation during the cell proliferation phase. Suppressing those later developmental processes may be important for cell proliferation activity because as seen in cell-size homeostasis phenomenon, cell proliferation and cell expansion/differentiation are often temporarily separated. In the following part, we will discuss candidate pathways.

### 4.2 Gibberellin is a candidate factor downstream of AN3 to regulate the transition from cell proliferation to expansion

Based on comparison with previous transcriptome datasets, we identified a candidate gene associated with precocious cell differentiation in *an3* cells. *NPF3* (AT1G68570), which encodes a membrane-localized GA transporter, was upregulated in the *an3* mutant (**Supplementary Figure 5**, **Table S2**). The *npf3* mutant is defective in GA accumulation in elongating endodermal cells, and ectopic expression of *NPF3* increases GA accumulation in various root cell types (Tal et al., 2016). Therefore, upregulated *NPF3* in the *an3* mutant may increase the uptake of GA by *an3* proliferating cells.

The relationship between GA and the phase transition from proliferation to expansion has been demonstrated during maize leaf development. GA accumulates around the transition zone in maize leaves, making a narrow peak around the boundary between the division zone (DZ) and the elongation zone (EZ); the accumulated GA is immediately catabolized in the EZ. Increased or decreased GA levels in leaves changed the position of the GA peak closer to the base or distal to the leaf primordia, respectively, resulting in a longer or shorter DZ (Nelissen et al., 2012). This indicates that the position of the GA accumulation peak regulates the transition from cell proliferation to expansion. It should be noted that the complex formed between ZmAN3 and ZmGRFs is also a key regulator of this phase transition. In the wild type, ZmAN3 changes its interacting GRFs in the DZ and EZ depending on the enrichment of each GRF. By changing these interactors, the AN3 complex regulates the transition from proliferation to expansion. For example, the overexpression of *ZmGRF1*, a positive regulator of cell division that interacts with ZmAN3 in the DZ, leads to a longer DZ (Nelissen et al., 2015). Thus, ZmAN3 and GA are significant regulators of the transition from cell proliferation to expansion.

Considering the role of ZmAN3 and GA in maize leaves, together with our data showing upregulated *NPF3* in the Arabidopsis *an3* mutant, we suggest that GA plays a role in regulating the phase transition from cell proliferation to expansion downstream of AN3, as well as in Arabidopsis leaf primordia. One possible scenario is that AN3 suppresses the expression of *NPF3*, and in the absence of AN3, the upregulation of *NPF3* leads to increased cellular uptake of GA into the cell. Similar to maize leaves, accumulated GA may cause a transition from cell proliferation to expansion. Our results suggest a novel role of AN3 and GA through NPF3 in the transition regulation of Arabidopsis leaf development.

### 4.3 Cytokinin is another candidate involved in the precocious onset of cell differentiation in the *an3* mutant

A previous study has shown that CK levels in Arabidopsis leaf primordia changes dynamically. The level of *t*Z, the active form of CK, is high when all cells proliferate in the leaf primordia and rapidly decreases after the onset of cell expansion (Skalák et al., 2019). They have shown that only *LOG3*, which encodes a cytokinin-activating enzyme, alters the expression levels among CK biosynthesis genes. Its expression level is high when all cells are proliferating and decreases before the onset of cell expansion. Other *LOG*s and *IPT*s do not significantly alter the expression levels. In addition, if CK-degradation is induced during the proliferation phase, smaller leaves with fewer and larger cells are produced. This is caused by a premature transition from the cell proliferation phase and a precocious acceleration of cell expansion and differentiation (Holst et al., 2011; Skalák et al., 2019). These phenotypes resembled those of the *an3* mutant. Our RNA-seq analyses showed that *LOG3* expression was significantly lower in the *an3* mutant than in the wild type (**Figure 4C**). Altered expression of *AHK4* and *ARR4* also suggested that the CK response in *an3* proliferating cells was similar to that in wild-type expanding cells. In addition, *ARR4* is directly suppressed by AN3 (Vercruyssen et al., 2014). *ARR4* is a type-A *ARR*, a negative regulator of CK signaling (To et al., 2007), suggesting that *ARR4* expression in wild type is suppressed during the cell proliferation phase while its suppression is released in the *an3* mutant, leading to the repression of CK signaling. Antagonistic interactions among type-A ARRs have been also suggested (To et al., 2007), therefore, upregulated *ARR4* may not directly lead to CK deficiency. However, the similarity in the phenotypes between the *an3* mutant and the CK-deficient line strongly suggests that CK levels or signaling are decreased in the *an3* proliferating cells.

CK is well known to play a role in promoting chloroplast development (reviewed by Cortleven and Schmülling, 2015). If CK level or signaling is decreased in *an3*, the accelerated chloroplast development in the *an3* proliferating cells was seemingly opposite phenotype. However, the effect of CK on chloroplast development differed depending on developmental timing. Previous study has shown that CK deficiency in proliferating cells does not change the final chlorophyll fluorescence levels in mature leaves (Skalák et al., 2019). Another study has shown that the chloroplast number per cell increases in the *an3* mutant, making the chloroplast density in a cell comparable to that of the wild type (Kawade et al., 2013). Thus, the accelerated chloroplast development in the *an3* proliferating cells could result in more chloroplasts but it does not change the final density of chloroplasts per a mature cell. The phenotypes we observed in the *an3* mutant indicated that the CK level would be decreased in *an3* proliferating cells compared to that in the wild type and that decreased CK level could be involved in the precocious activation of cell differentiation in *an3*.

In the present study, we found that *an3* mutant leaf primordia showed precocious cell expansion, chloroplast development, vacuole development, and cell wall composition alterations during the cell proliferation phase. These results, together with the RNA-seq data, suggested that AN3 plays a role in suppressing cell differentiation during the cell proliferation phase. This is a novel role for AN3, which is known to be a positive cell proliferation regulator. Because precocious cell differentiation in the *an3* mutant was observed with active cell proliferation, AN3 plays a dual role in regulating phase transition and cell proliferation. Moreover, our results suggested that GA and CK are involved in regulating the transition from cell proliferation to expansion downstream of AN3 during Arabidopsis leaf development.

## Data availability statement

The datasets presented in this study can be found in online repositories. The data presented in the study are deposited in the DDBJ Sequence Read Archive, the run accession number DRR506123–DRR506128.

## Author contributions

KE: Data curation, Formal analysis, Investigation, Validation, Visualization, Writing – original draft, Writing – review & editing. HK: Data curation, Formal analysis, Investigation, Writing – review & editing. NT: Data curation, Formal analysis, Writing – review & editing. KT: Data curation, Formal analysis, Writing – review & editing. TH: Data curation, Validation, Writing – review & editing. SS: Data curation, Formal analysis, Writing – review & editing. HT: Funding acquisition, Supervision, Writing – review & editing.
