# Supplementary material for: Precocious cell differentiation occurs in proliferating cells in leaf primordia in Arabidopsis angustifolia3 mutant
Source: Front Plant Sci. 2024 Apr 16;15:1322223. doi: 10.3389/fpls.2024.1322223 (PMC11058843; doi:10.3389/fpls.2024.1322223)
Supplement: Supplementary file 1 [file DataSheet_1.docx]

Supplementary Material


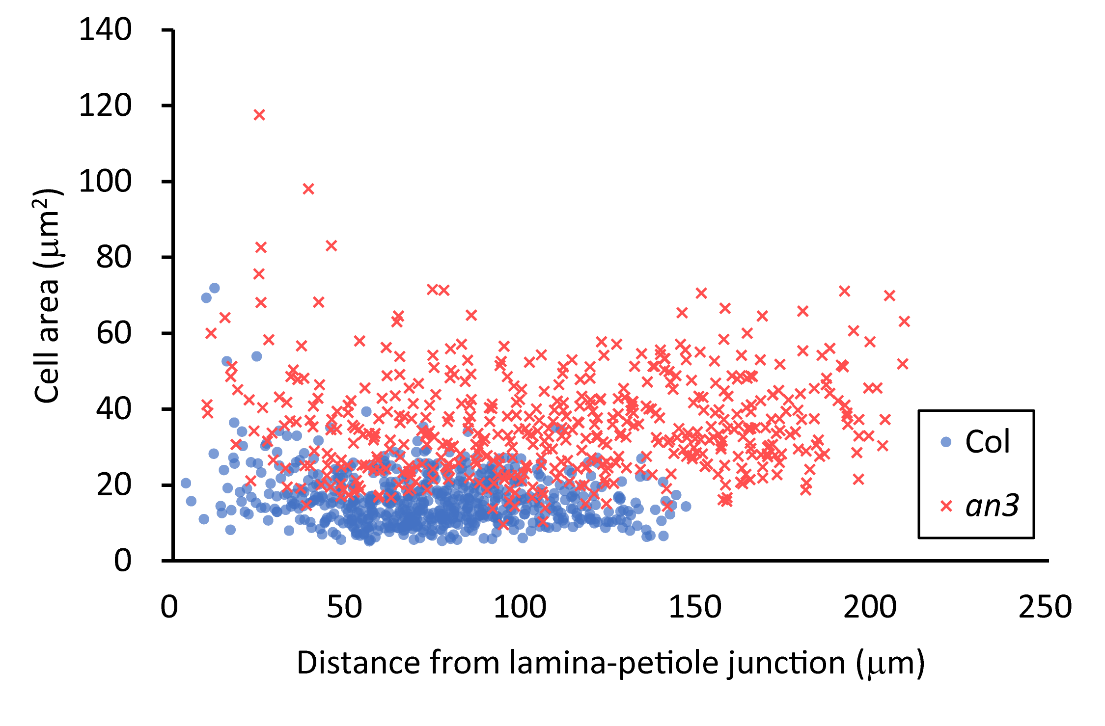


**Supplementary Figure 1.** Cell area distribution in 5 DAS leaf primordia. Each dot shows the cell area of wild type (blue) and *an3* cells (red) plotted against the distance from a junction between the leaf blade and petiole. n = 5 leaves for each line.


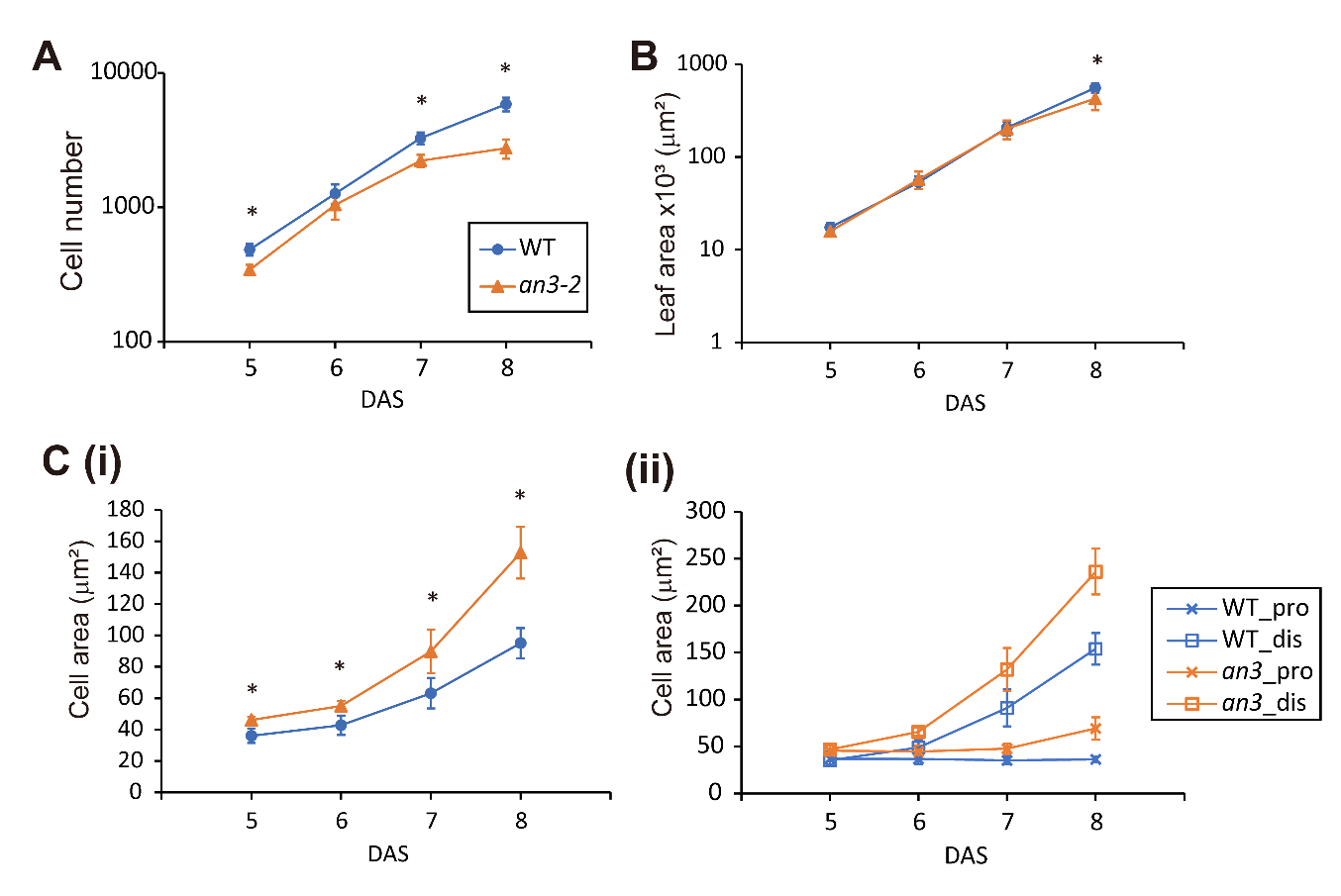


**Supplementary Figure 2.** Kinematic analysis of leaf primordia of wild type (circle, blue) and the *an3-2* mutant (triangle, orange). (A-C) Cell number (A), leaf area (B), cell area (C) from first foliage leaf primordia. In C, (i) shows average cell area and (ii) shows the proximal (cross mark) and distal (open square) cell areas, separately. Mean ± SD. n = 85–90 cells from 6 individuals were measured (* p < 0.01, Welch’s t-test for A and Wilcoxson rank sum test for B and C).

　　　
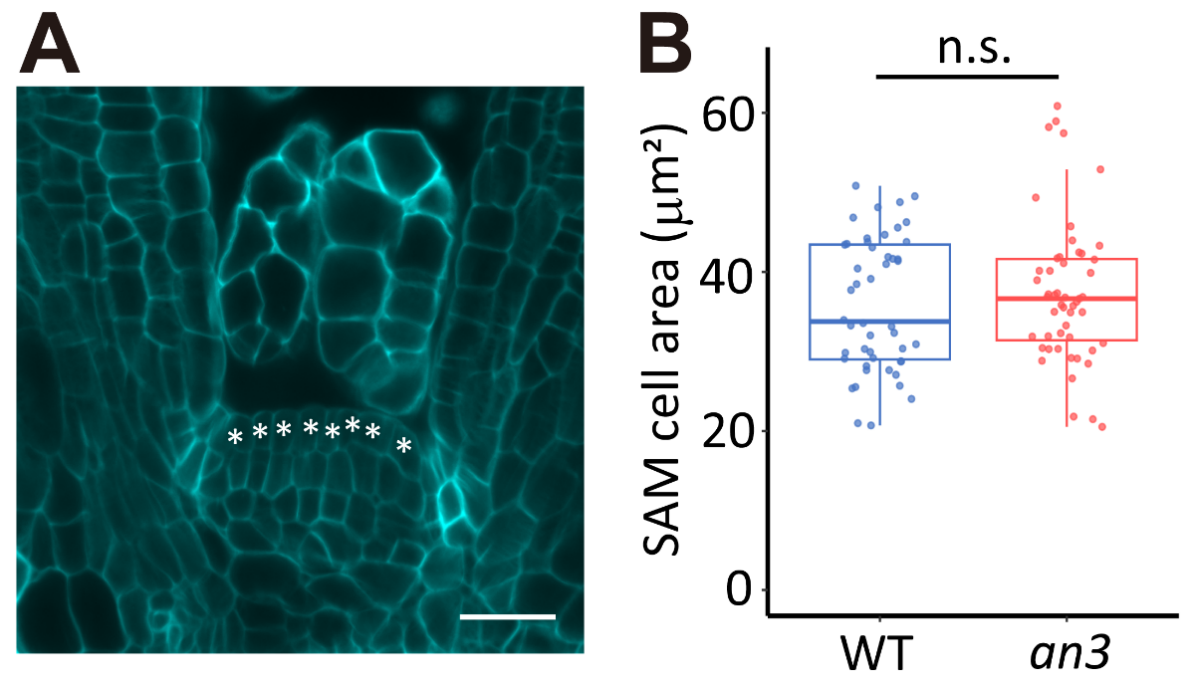


**Supplementary Figure 3.** Cell area in the shoot apical meristem (SAM). (A) A representative image of wild type SAM on 5 DAS. Asterisks indicate where cell area was measured. Cell wall was stained by calcofluor. Bar = 20 μm. (B) SAM cell area in WT and *an3* on 5 DAS. Each dot shows the cell area of wild type (blue) and *an3* cells (red) (n = 48 (WT) and 52 (*an3*) cells from 6 individuals. n.s., not significant; Wilcoxson rank sum test).


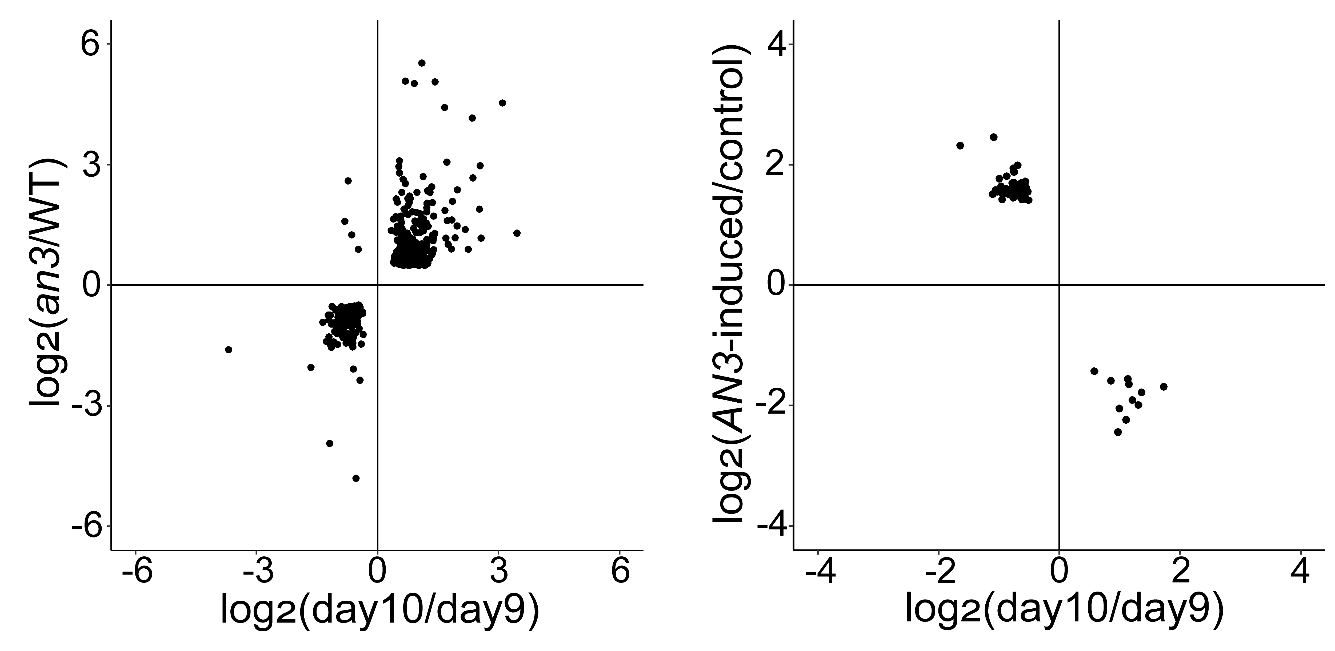


**Supplementary Figure 4.** Comparison of gene expression changes. Log value ratio of normalized gene expression levels was plotted. Each dot indicates a gene. (A) The 396 common genes between *an3* DEGs (5 DAS 1st foliage leaf primordia, *an3* mutant compared to wild type; the present analysis) and transition phase DEGs (wild type 3rd foliage leaf primordia, day 10 compared to day 9; Andriankaja et al. 2012). Spearman's rank correlation: ρ = 0.86. (B) The 59 common DEGs between *AN3*-induced DEGs (8 DAS 1st leaf primordia, 8-hour after AN3 induction compared with control; Vercruyssen et al. 2014) and transition phase DEGs (wild type 3rd foliage leaf primordia, day 10 compared to day 9; Andriankaja et al. 2012). Spearman's rank correlation: ρ = -0.56.


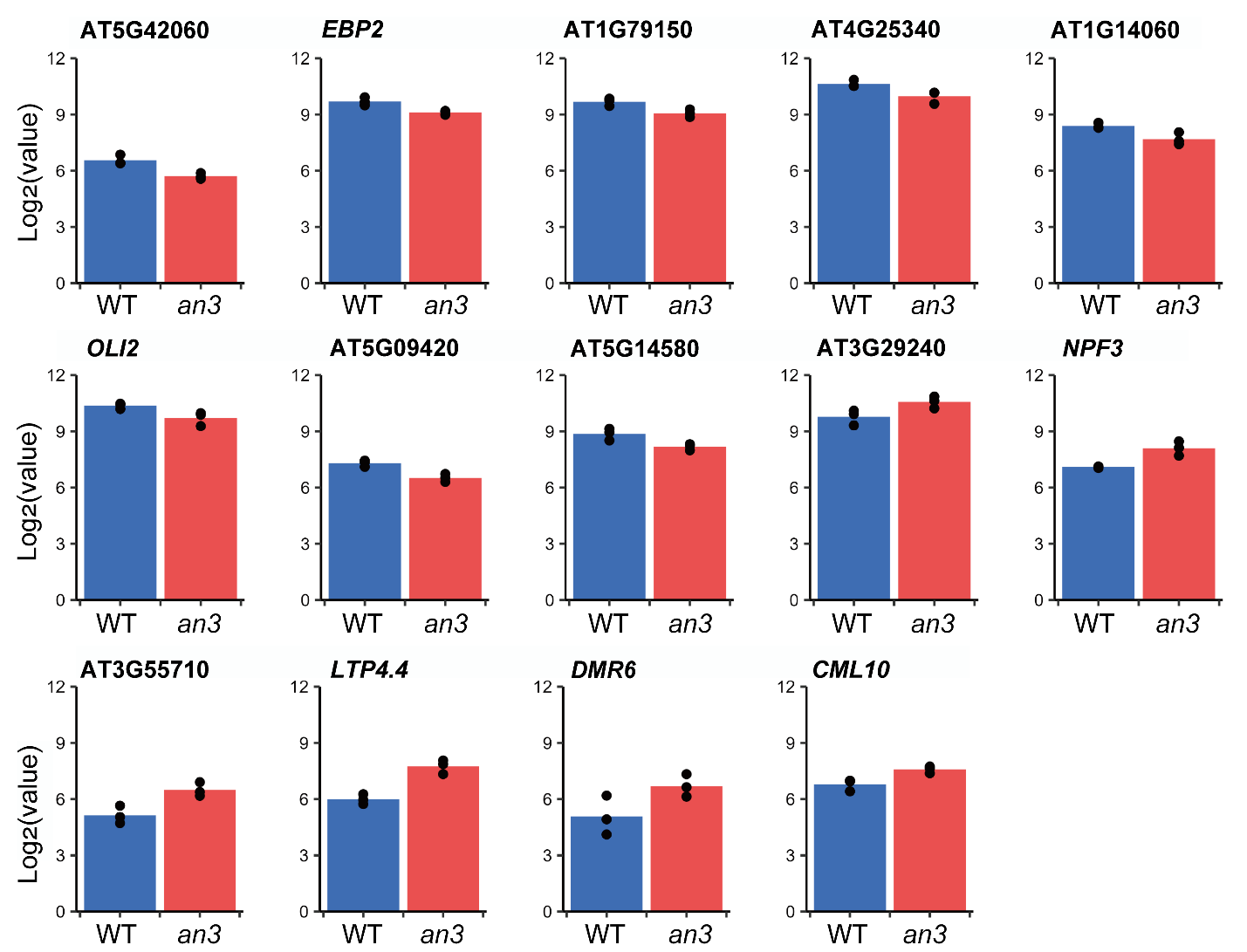


**Supplementary Figure 5.** Gene expression of 14 common genes, which are from the DEGs comparison, in WT (blue) and *an3* (red) 5 DAS leaf primordia. Log-transformed normalized counts are shown. Each dot shows replicates.
